# Prognostic value of plasma circulating tumor DNA fraction across four common cancer types: a real-world outcomes study^☆^

**DOI:** 10.1016/j.annonc.2022.09.163

**Published:** 2022-10-05

**Authors:** Z. R. Reichert, T. M. Morgan, G. Li, E. Castellanos, T. Snow, F. G. Dall’Olio, R. W. Madison, A. D. Fine, G. R. Oxnard, R. P. Graf, D. G. Stover

**Affiliations:** 1 University of Michigan, Ann Arbor; 2 Foundation Medicine, Cambridge; 3 Flatiron Health, New York, USA; 4 Gustave Roussy, Villejuif, France; 5 University of Bologna, Bologna, Italy; 6 The Ohio State University, Columbus, USA

**Keywords:** prostate cancer, breast cancer, non-small-cell lung cancer, colorectal cancer, tumor fraction, prognosis

## Abstract

**Background:**

Genomic analysis of circulating tumor DNA (ctDNA) is increasingly incorporated into the clinical management of patients with advanced cancer. Beyond tumor profiling, ctDNA analysis also can enable calculation of circulating tumor fraction (TF), which has previously been found to be prognostic. While most prognostic models in metastatic cancer are tumor type specific and require significant patient-level data, quantification of TF in ctDNA has the potential to serve as a pragmatic, tumor-agnostic prognostic tool.

**Patients and methods:**

This study utilized a cohort of patients in a nationwide de-identified clinico-genomic database with metastatic castration-resistant prostate cancer (mCRPC), metastatic breast cancer (mBC), advanced non-small-cell lung cancer (aNSCLC), or metastatic colorectal cancer (mCRC) undergoing liquid biopsy testing as part of routine care. TF was calculated based on single-nucleotide polymorphism aneuploidy across the genome. Clinical, disease, laboratory, and treatment data were captured from the electronic health record. Overall survival (OS) was evaluated by TF level while controlling for relevant covariables.

**Results:**

A total of 1725 patients were included: 198 mCRPC, 402 mBC, 902 aNSCLC, and 223 mCRC. TF ≥10% was highly correlated with OS in univariable analyses for all cancer types: mCRPC [hazard ratio (HR) 3.3, 95% confidence interval (CI) 2.04–5.34, *P* < 0.001], mBC (HR 2.4, 95% CI 1.71–3.37, *P* < 0.001), aNSCLC (HR 1.68, 95% CI 1.34–2.1, *P* < 0.001), and mCRC (HR 2.11, 95% CI 1.39–3.2, *P* < 0.001). Multivariable assessments of TF had similar point estimates and CIs, suggesting a consistent and independent association with survival. Exploratory analysis showed that TF remained consistently prognostic across a wide range of cutpoints.

**Conclusions:**

Plasma ctDNA TF is a pragmatic, independent prognostic biomarker across four advanced cancers with potential to guide clinical conversations around expected treatment outcomes. With further prospective validation, ctDNA TF could be incorporated into care paradigms to enable precision escalation and de-escalation of cancer therapy based on patient-level tumor biology.

## INTRODUCTION

Cancer ‘liquid biopsy’ is an increasingly adopted diagnostic approach for querying the biology of a cancer through minimally invasive analysis of the blood.^1^ Early liquid biopsy approaches focused on analysis of circulating tumor cells (CTCs); while CTCs may represent the metastatic potential of a cancer, they are absent in many metastatic patients [up to 80% of stage III/IV non-small-cell lung cancer (NSCLC) patients have no circulating CTCs],^2–4^ and low detection levels can minimize the dynamic range of the test.^5^ More recently, circulating tumor DNA (ctDNA) analysis has emerged with widespread clinical adoption and regulatory approval of multiple multi-gene assays.^6,7^ Levels of ctDNA shed by a given cancer can be variable over time and can be influenced by cancer stage and therapy, such that a negative liquid biopsy does not rule out the presence of an actionable alteration on tumor tissue analysis.

Liquid biopsy is increasingly incorporated into clinical practice guidelines for the management of late-stage cancer patients.^8,9^ These ctDNA analyses are variable in their design, with some focused on a single gene alteration and others more comprehensively sequencing the cancer genome.^9,10^ One previously described technological opportunity from broad genomic analysis of ctDNA is in the quantification of ctDNA shed by measuring tumor fraction (TF). Stover et al. measured TF in patients with metastatic breast cancer (mBC) through quantifying aneuploidy across the genome and found that TF ≥10% was prognostic independent of clinicopathological factors in a multivariate analysis [hazard ratio (HR) 2.14, 95% confidence interval (CI) 1.4–3.8, *P* < 0.001].^11^ Both Kohli et al. and Choudhury et al. have described higher TF as portending a worse prognosis in hormone-sensitive and/or metastatic castration-resistant prostate cancer (mCRPC).^12,13^ To date, most prognostic implications of TF quantification have been evaluated primarily within individual disease settings and not across cancer types.

Clinical determination of patient-level prognosis is a well-established paradigm for informing decision making. In the curable setting, TNM (tumor–node–metastasis) staging is dominant, and cancer type specific, with gradual integration of molecular correlates for some cancer types. For patients with metastatic or advanced disease, prognostic stratification typically incorporates both disease factors (tumor burden, molecular correlates, response to prior therapies) and patient factors (comorbidities, performance status). The availability of validated approaches to risk stratification varies by disease. For some cancers, disease-specific nomograms have been developed to integrate patient-level prognostic factors,^14–16^ but have variable adoption. Here, we study whether the quantification of ctDNA shed using a TF biomarker, based on widely available commercial liquid biopsy testing, could offer robust prognostic information for patients with advanced cancer across multiple tumor types.

## PATIENTS AND METHODS

### Study design and patients

This study used the nationwide (US-based) de-identified Flatiron Health-Foundation Medicine clinico-genomic database (CGDB). The de-identified data originated from ~280 US cancer clinics (~800 sites of care). Retrospective longitudinal clinical data were derived from electronic health record (EHR) data, comprising patient-level structured and unstructured data, curated via technology-enabled abstraction, and were linked to genomic data derived from Foundation Medicine, Inc comprehensive genomic profiling (CGP) tests in the CGDB by de-identified, deterministic matching.^17^ Institutional review board (IRB) approval of the study protocol by the WCG IRB (registration number IRB00000533) was obtained before study conduct, and included a waiver of informed consent. Data cut-off date was 30 June 2021.

**Figure 1.**
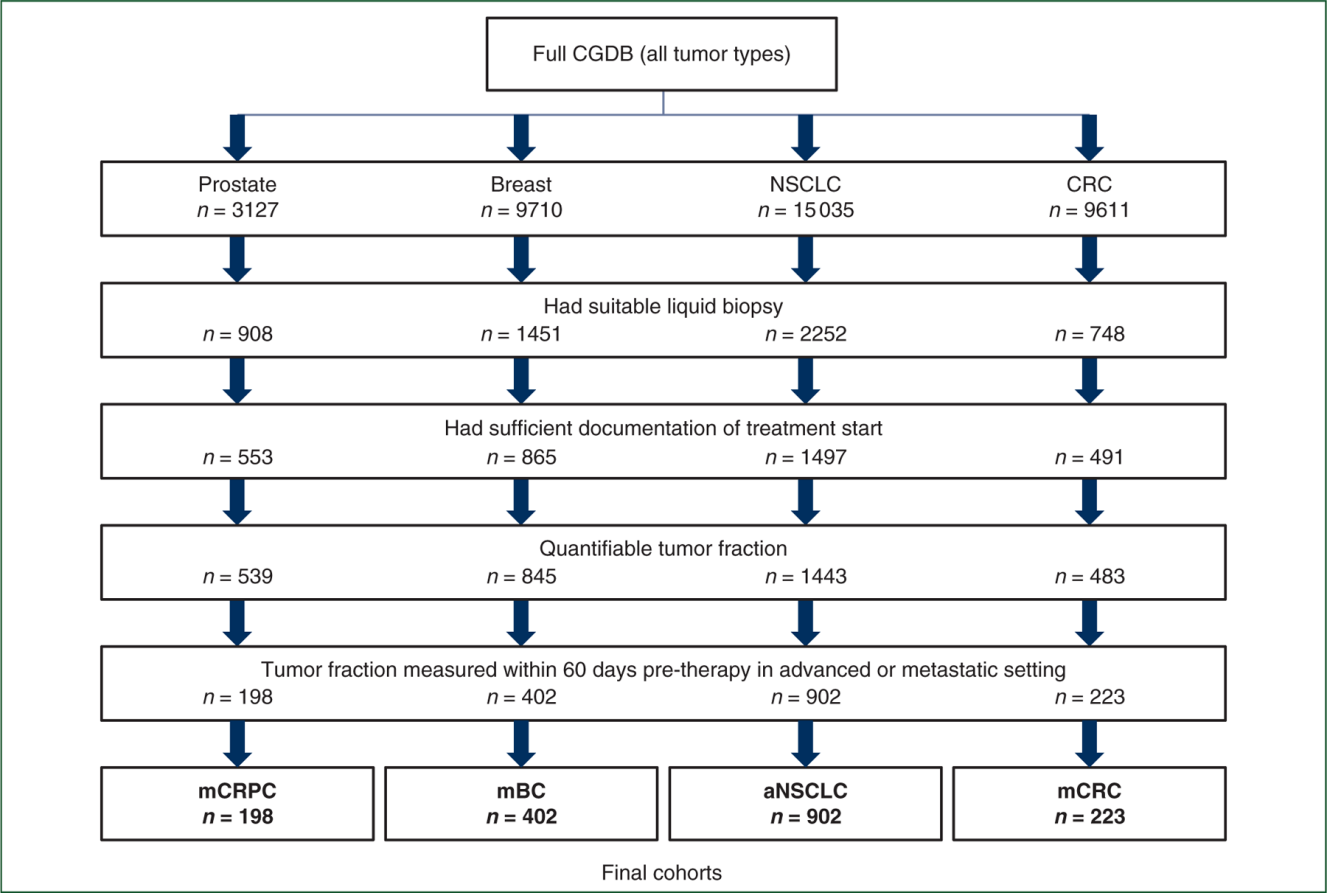
Consolidated Standards of Reporting Trials (CONSORT) diagram showing cohort attrition after applying inclusion and exclusion criteria to the four datasets in the tumor types of interest. aNSCLC, advanced non-small-cell lung cancer; CGDB, clinico-genomic database; CRC, colorectal cancer; mBC, metastatic breast cancer; mCRC, metastatic colorectal cancer; mCRPC, metastatic castration-resistant prostate cancer; NSCLC, non-small-cell lung cancer.

The study leveraged a multi-tumor cohort, which included patients with mCRPC, mBC, advanced (stage IIIB-IV or progressive/recurrent) NSCLC (aNSCLC), and metastatic colorectal cancer (mCRC). All cancer diagnoses, including metastatic/advanced status, were confirmed via review of patient charts. Eligibility for inclusion in this study among the CGDB multi-tumor cohort is outlined in the Consolidated Standards of Reporting Trials (CONSORT) diagram (Figure 1). Patients in the final study cohort needed to have been tested with FoundationOne^®^ Liquid or FoundationOne^®^ Liquid CDx (Foundation Medicine, Cambridge, MA) on a specimen collected within 60 days before the start of a systemic line of therapy and this test must have resulted in a quantifiable (i.e. a single, discrete number, which may be zero) TF result. Most patients in the CGDB completed tissue-based Foundation Medicine testing, which led to 14.3% (5359/37 483) of total patients across cancer types having suitable liquid biopsy. Lines of therapy were derived from structured and unstructured EHR treatment data using an oncologist-defined, rule-based approach^18^ and counted only systemic lines of therapy within the metastatic/advanced setting. Patients must also have structured EHR activity within 90 days of their advanced/metastatic diagnosis date to optimize completeness of treatment data capture and improve accuracy in the enumeration of their lines of therapy. For patients with multiple TF results across different lines of therapy, the earliest line of therapy and the temporally closest TF to the therapy start date was used.

### Comprehensive genomic profiling

Hybrid capture-based next-generation sequencing (NGS) was carried out as a part of routine clinical care (Foundation Medicine). The 70-gene FoundationOne^®^ Liquid assay and the 324-gene FoundationOne^®^ Liquid CDx assay^19^ assess base substitutions, short insertions/deletions, rearrangements/fusions, and copy number variations.

The levels of ctDNA shed for each specimen was quantified by calculating an investigational composite TF,^20^ which merges two methods for estimation of TF.^21^ When TF is elevated (generally >10%), an estimate is returned based on measure of tumor aneuploidy that incorporates observed deviations in coverage across the genome.^22^ This aneuploidy-based approach avoids erroneously inferring elevated TF due to the presence of germline variants detected at high variant allele frequency. When lack of tumor aneuploidy limits the ability to estimate TF (generally at lower TF), a variant-based calculation is made by identifying the highest allele fraction non-germline variant, excluding specific clonal hematopoiesis-associated alterations. The primary analyses of this study treated TF as a binary variable, indicating whether a specimen had TF ≥10% or TF <10%. This cutpoint was selected to align with previous work by Stover et al.^11^ Exploratory analyses assessed the effect of varying this cutpoint.

### Clinicopathological covariables

The prognostic value of TF was assessed within the context of other known clinicopathological covariables. Due to differences in established prognostic models among different diseases, we selected a panel of covariates specific for each type of cancer. Variables were selected by expert consultation based on a combination of documented prognostic relevance in the tumor type and availability in the CGDB. The selected variables are presented in Table 1. All variables were measured within the same 60-day pre-therapy window as TF except: hormone receptor and human epidermal growth factor receptor 2 statuses in breast counted any pre-therapy positive result as positive for the patient, sites of metastasis counted all metastases detected pre-therapy, sidedness of CRC was indexed to initial diagnosis using a previously described International Classification of Diseases-based approach.^23^

### General statistical considerations

Missing values in variables with <20% missingness were imputed with simple imputation with the expected values conditional on observed covariates using random forests with the R package ‘missForest’, and these imputed values were treated identically to measured values in a subsequent analysis. Variables with greater degrees of missingness had missing values treated as a separate category. Overall survival (OS) was calculated from the start of treatment to death from any cause, and patients alive at last observation were right censored. The CGDB is built on a dataset that is at least 85% sensitive for detecting patient deaths when benchmarked against the National Death Index.^24^ Issuance of an Foundation Medicine, Inc liquid biopsy CGP report was an inclusion criterion for the database used in this study, and this can potentially occur after the start of therapy. As a result, this dataset is left truncated for the purposes of OS analyses. To account for this, risk-set adjustment was carried out, including only patients who have met all inclusion criteria at each time point as at risk in Kaplan–Meier and Cox model analyses.^25^ The assumption of independent left truncation was verified by univariable modeling of the effect of delayed entry time on the survival outcome. R version 4.2.1 software (R Foundation for Statistical Computing, Vienna, Austria) was used for all statistical analyses.

### Statistical analyses

The prognostic value of TF was assessed in both univariable and multivariable contexts. The primary univariable analysis consisted of Kaplan–Meier plots of OS for each tumor type stratified by TF at a cutpoint of 10% and corresponding Cox proportional hazards models. Median OS and HRs with their respective 95% CIs, along with log-rank *P* value, are all reported.

The primary multivariable analysis consisted of a single multivariable Cox proportional hazards model per tumor type that incorporates TF at a cutpoint of 10% and all the variables listed in Table 1. From this, a forest plot showing HRs and *P* values for each variable level was generated per tumor type.

An exploratory analysis was conducted to assess the effect of varying the TF cutpoint used in the primary analysis. TF values between 1% and 20% were tested at 1% increments and the HR for the univariable Cox proportional hazards model of OS was plotted for each cutpoint.

## RESULTS

### Patient population

The selection process yielded 1725 total patients: 198 mCRPC, 402 mBC, 902 aNSCLC, 223 mCRC (Figure 1). These numbers represent 21.8%, 27.7%, 40.0%, and 29.8%, respectively, of the patients who had a suitable liquid biopsy in each cancer type. The liquid biopsy specimen collection date ranges per disease were 12 September 2018 to 17 June 2021 for mCRPC, 17 September 2018 to 15 June 2021 for mBC, 17 September 2014 to 18 June 2021 for aNSCLC, and 4 February 2018 to 16 June 2021 for mCRC. The patient characteristics (separated by TF as well) of each cancer type reflect the expected disease traits (Supplementary Table S1, available at https://doi.org/10.1016/j.annonc.2022.09.163). The cohort also included cancers with a mix of molecular subgroups [16% epidermal growth factor receptor (EGFR)-positive aNSCLC, 48% RAS-positive mCRC] and sites of disease (e.g. bone, liver, brain). No variable used in the analysis had >20% missingness before imputation.

### High and low TF is robustly associated with prognosis across cancer types

**Figure 2.**
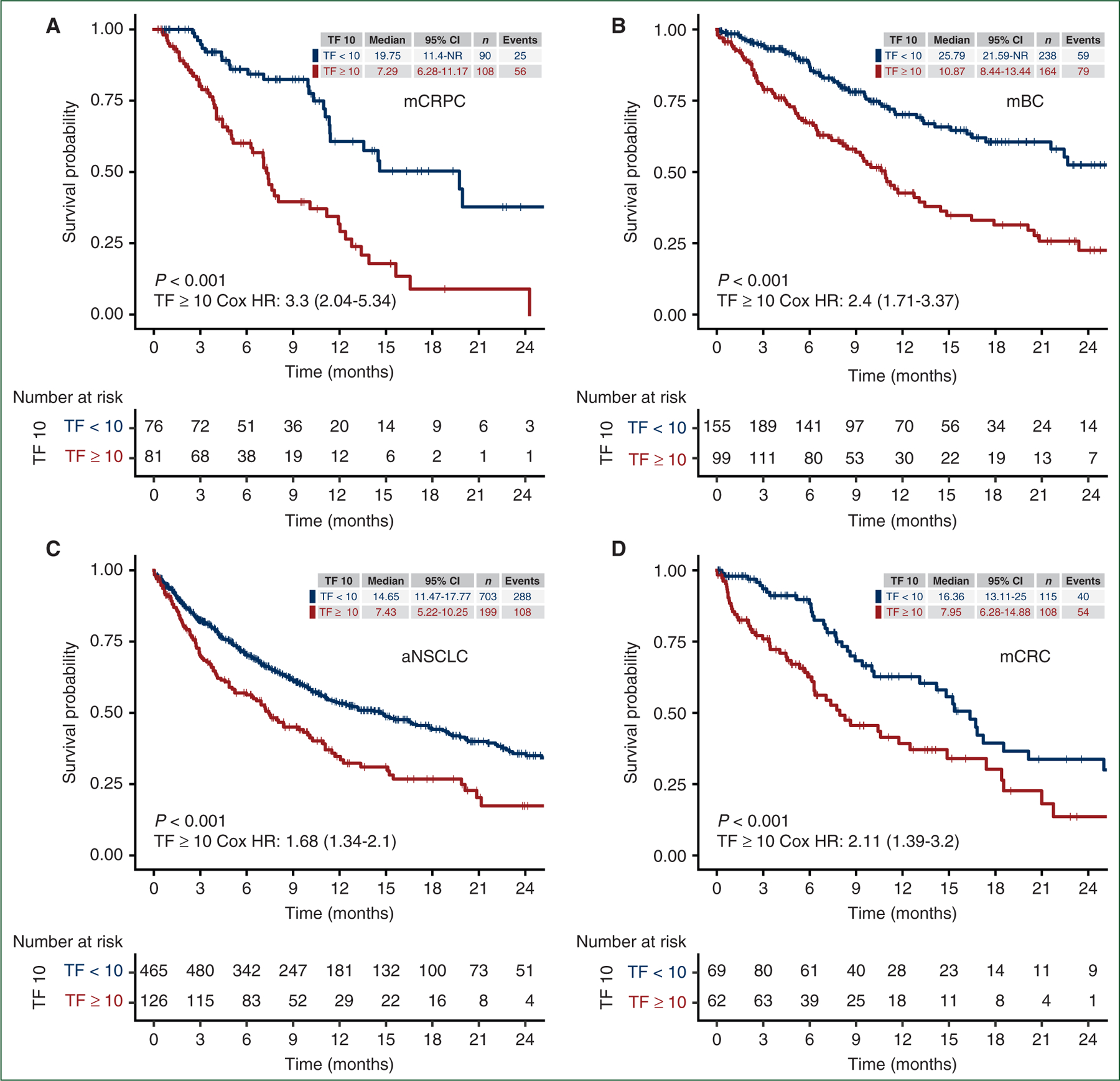
Elevated TF is prognostic for worse overall survival in the four tumor histologies studied. Kaplan–Meier plots of real-world overall survival from therapy start, stratified by tumor fraction at a cut-off of 10% as measured within 60 days prior, in (a) mCRPC, (b) mBC, (c) aNSCLC, and (d) mCRC. aNSCLC, advanced non-small-cell lung cancer; CI, confidence interval; HR, hazard ratio; mBC, metastatic breast cancer; mCRC, metastatic colorectal cancer; mCRPC, metastatic castration-resistant prostate cancer; NR, not reached; TF, tumor fraction.

Across cancer types, elevated TF of at least 10% was strongly associated with worse OS in univariable analyses (Figure 2). Compared to those with TF <10%, those with TF ≥10% had a greater hazard death in each cancer type: mCRPC (HR 3.3, 95% CI 2.04–5.34, *P* < 0.001), mBC (2.4, 95% CI 1.71–3.37, *P* < 0.001), aNSCLC (1.68, 95% CI 1.34–2.1, *P* < 0.001), and mCRC (2.11, 95% CI 1.39–3.2, *P* < 0.001).

**Figure 3.**
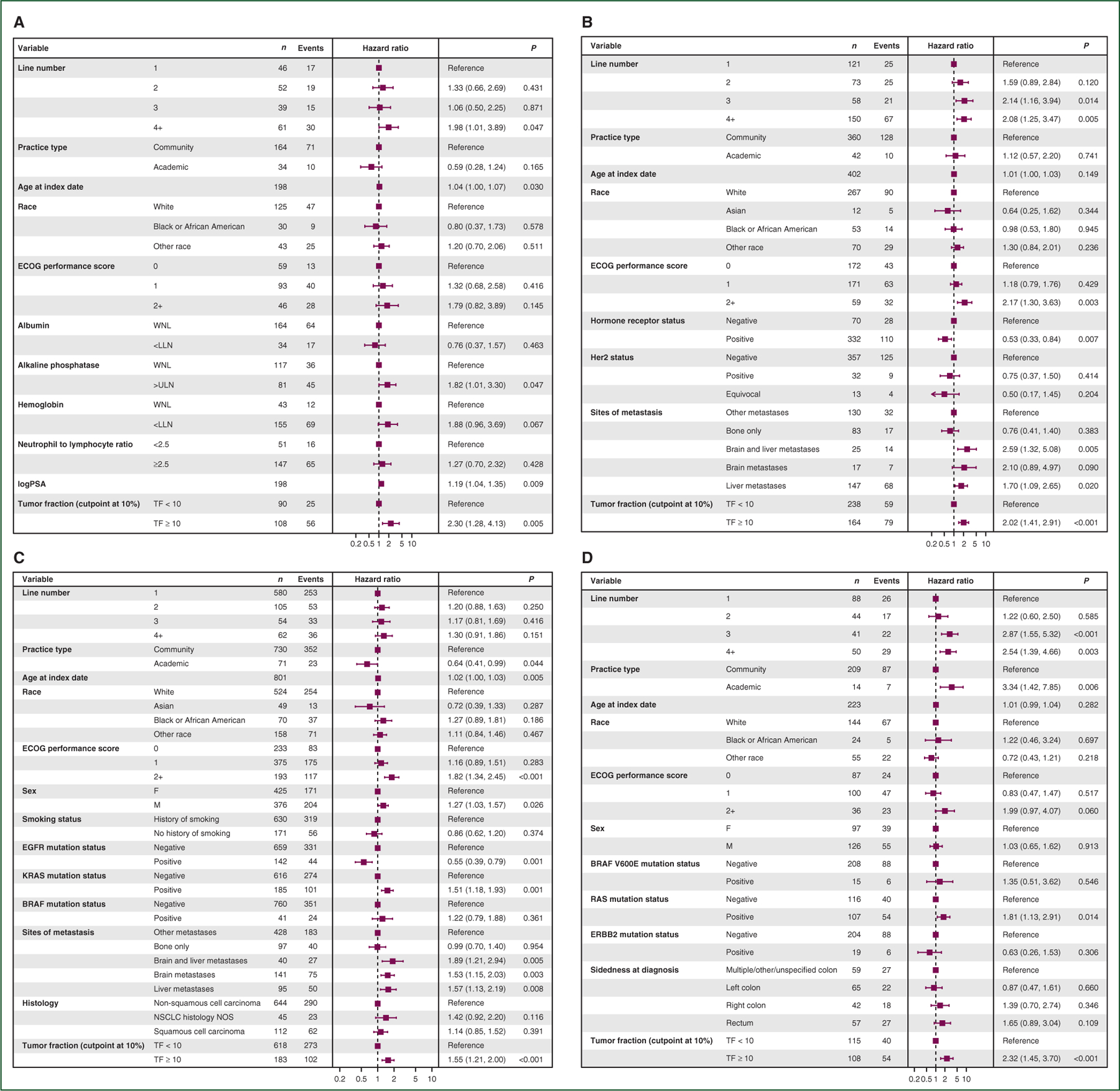
Multivariable modeling shows elevated TF remains highly prognostic for worse overall survival across tumor types even after adjusting for established disease-specific prognostic markers. Forest plots showing hazard ratios for each variable used in multivariable Cox proportional hazards modeling for (a) mCRPC, (b) mBC, (c) aNSCLC, and (d) mCRC. aNSCLC, advanced non-small-cell lung cancer; ECOG, Eastern Cooperative Oncology Group; LLN, lower limit of normal; mBC, metastatic breast cancer; mCRC, metastatic colorectal cancer; mCRPC, metastatic castration-resistant prostate cancer; TF, tumor fraction; ULN, upper limit of normal; WNL, within normal limits.

Because patient characteristics can be highly heterogeneous, we sought to evaluate if the presence of at least 10% TF had independent prognostic value to standard clinical and pathological features utilized for assessment of patient prognosis. The extracted data are incomplete to directly compare with other validated full-risk models, reflecting those models’ intrinsic complexity. A good faith effort was made to extract as many features as possible (see Patients and methods). The adjusted point estimates for OS with CIs for TF of 10% were similar to the unadjusted estimates (Figure 3). The HR for death was 2.30 for mCRPC (95% CI 1.28–4.13, *P =* 0.005), 2.02 for mBC (95% CI 1.41–2.91, *P* < 0.001), 1.55 for aNSCLC (95% CI 1.21–2.00, *P* < 0.001), and 2.32 for mCRC (95% CI 1.45–3.70, *P* < 0.001). TF is an independent risk factor after adjusting for differences in the evaluated clinical features.

### TF is less prognostic for aNSCLC patients with EGFR+ or those with brain metastases

Because patients with aNSCLC represented the majority of the population analyzed, additional analysis of prognostic subgroups was carried out on the aNSCLC cohort (Supplementary Figure S1, available at https://doi.org/10.1016/j.annonc.2022.09.163). When stratifying aNSCLC patients by EGFR mutation status, elevated TF of at least 10% was found to be directionally less prognostic for OS in patients whose tumors were EGFR+ by NGS [HR 1.46 (0.78–2.71)] versus EGFR− [HR 1.94 (1.52–2.46)], although the CIs of the HRs are wide and overlapping. With availability of highly effective initial targeted therapy options for EGFR+ disease, fewer deaths happen within the first year for high TF disease, such that more patients and longer follow-up may be needed to measure the true prognostic effect. Focusing on aNSCLC patients with or without brain metastases detected at any point before therapy, elevated TF was not prognostic for OS in patients with brain metastases [HR 1.1 (0.71–1.7)], while it still was prognostic for patients without brain metastases [HR 1.89 (1.46–2.45)]. Brain metastases tend to be highly prognostic in aNSCLC, yet are not themselves associated with ctDNA shed.^26,27^

### TF is prognostic across a range of cutpoints

**Figure 4.**
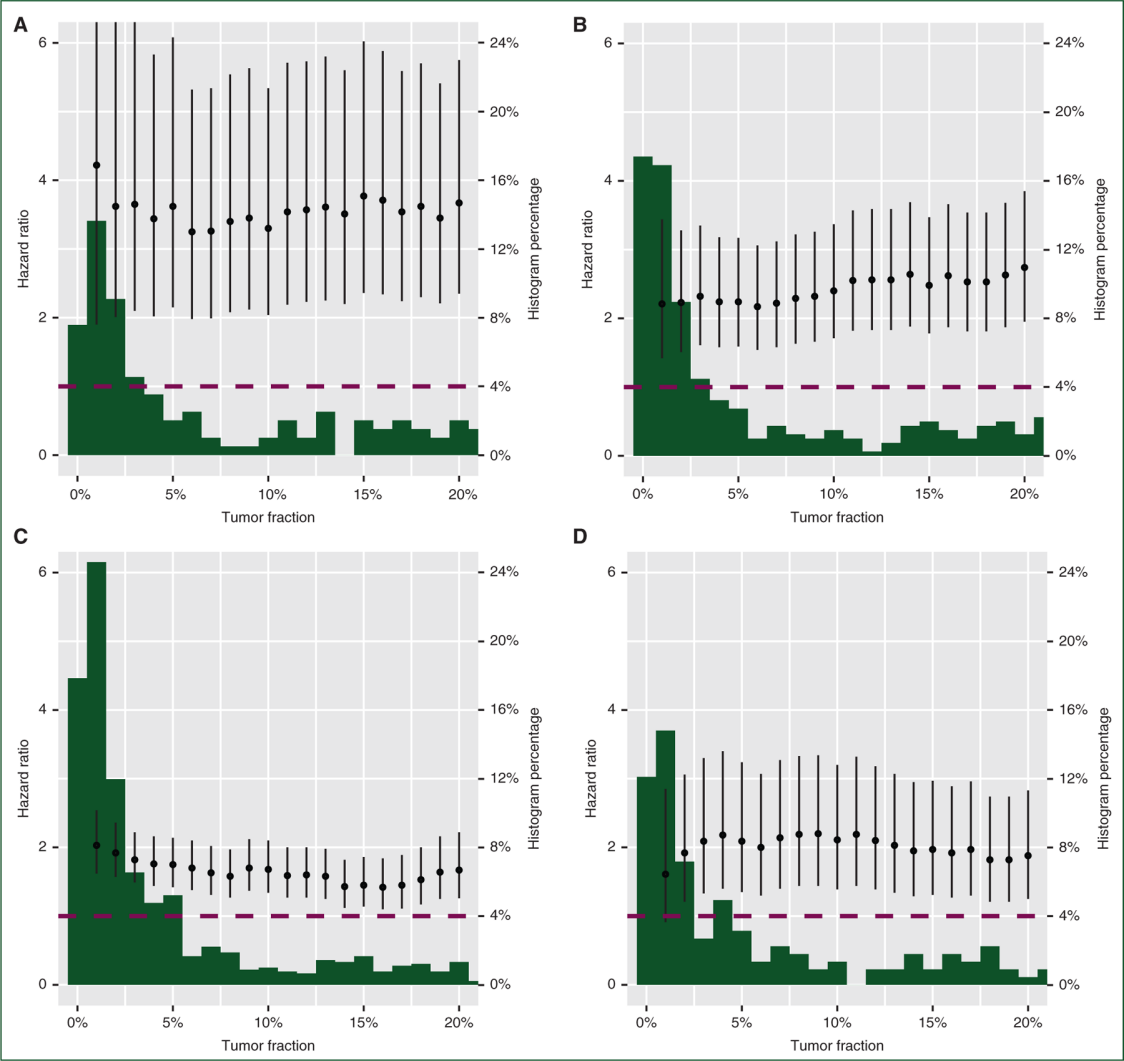
Exploratory analysis of varying the tumor fraction cutpoint shows tumor fraction remains prognostic for real-world overall survival across tumor types regardless of the cutpoint. Exploratory analysis of the effect of varying the cTF cutpoint on the hazard ratio for the TF high versus TF low groups in univariable Cox proportional hazards models. Cutpoints between 1% and 20% are tested in increments of 1% in (a) mCRPC, (b) mBC, (c) aNSCLC, and (d) mCRC. The dotted line shows a hazard ratio of 1. Behind each plot of hazard ratios is a histogram of TF values between 1% and 20%, expressed as a percentage of all patients per disease. The full histogram is presented in Supplementary Figure S2, available at https://doi.org/10.1016/j.annonc.2022.09.163. aNSCLC, advanced non-small-cell lung cancer; mBC, metastatic breast cancer; mCRC, metastatic colorectal cancer; mCRPC, metastatic castration-resistant prostate cancer.

The distribution of TF for each cancer type was plotted and range assessed (Supplementary Figure S2, available at https://doi.org/10.1016/j.annonc.2022.09.163). Across cancer types, TF displayed a heavily right-skewed distribution, not unlike the general characteristics of distributions of CTC enumerations.^28–30^ However, the specific ranges observed varied by cancer type; mCRPC had a median of 13% [interquartile range (IQR) 2%−31%], mBC had a median of 4% (IQR 1%−21%), aNSCLC had a median of 2% (IQR 1%−8%), and mCRC had a median of 8% (IQR 1%−38%). When looking at each cancer type individually, we find a consistent prognostic association with TF across a wide range of potential cutpoints from TF ± 1% to TF ± 20% with overlapping CIs (Figure 4). This suggests TF could be highly prognostic regardless of the exact cutpoint selected.

Finally, we explored whether low TF (<1%) could identify patients with a favorable prognosis across the cancer types studied (Supplementary Figure S3, available at https://doi.org/10.1016/j.annonc.2022.09.163). In mCRPC, 15% of patients had low TF and had a median OS of not reached (NR) (95% CI 19.75-NR) months; in mBC, 27% of patients had low TF and had a median OS of 25.79 (95% CI 22.67-NR) months; in aNSCLC, 33% of patients had a low TF and had a median OS of 22.51 (95% CI 19.25-NR) months; and in mCRC, 19% of patients had low TF and had a median OS of 15.34 (95% CI 10.02-NR) months. In mBC and aNSCLC, a 1% TF cut-off remained prognostically significant on multivariable analysis (Supplementary Figure S4, available at https://doi.org/10.1016/j.annonc.2022.09.163). A trichotomous analysis for each cancer type separating TF into TF <1%, 1%, ≤TF < 10%, and ≥10% showed a stepwise prognostic difference suggestive of a dose-dependent effect for mCRPC and aNSCLC, but less so for mBC and mCRC (Supplementary Figure S5, available at https://doi.org/10.1016/j.annonc.2022.09.163).

## DISCUSSION

In this analysis, we find that a single widely available blood-based biomarker (ctDNA TF) exhibits prognostic characteristics across cancer types in a US-based real-world dataset. The prognostic impact of TF is independent of most clinical features on multivariable analyses, thus offering orthogonal information. Interestingly, some dominant prognostic features dilute the impact of TF such as the presence of brain metastases in aNSCLC—brain metastases may not shed ctDNA and the morbidity of a small amount of brain disease can be catastrophic on its own. The reason that TF had less prognostic effect in EGFR+ aNSCLC could be due to the availability of highly effective systemic therapy for this patient cohort. Such findings were seen in a prior analysis of [^18^F]2-fluoro-2-deoxy-d-glucose–positron emission tomography scan to assess tumor burden in EGFR+ aNSCLC,^31^ which suggested that the reliable systemic effect from highly effective targeted therapies may overcome otherwise poor prognosis, though additional follow-up is needed to understand if TF impacts the eventual pattern and biology of resistance.

In mCRC, we observed in multivariable analysis that TF remains a significant prognostic factor alongside line number, practice type, and RAS mutation status. Notably, BRAF V600E mutation status and sidedness were not significant factors in our cohort. While we did not seek to review the contributions of all potential covariables, we selected the variables for this study on the basis of prior literature. The lack of signal from BRAF may be due to the small sample size (15 BRAF-positive patients). Similarly, over 20% of the mCRC cohort did not have a specific sidedness assigned on the basis of the data available. In this study, we followed the methodology outlined in Luhn et al. and while this approach is highly specific, it has known limitations compared to the gold standard of chart abstraction, including missingness for a sizable minority of patients, as has been previously reported.^23^ Along these lines, we are limited in the variables we can use in our multivariable analysis by the availability of data. Carcinoembryonic antigen is widely used to monitor response to therapy in mCRC and has been proposed as a pretreatment prognostic factor, but over 40% of the patients in our cohort did not have a measurement of this biomarker in the 60 days preceding the initiation of a new line of therapy.

An immediate application of these data could be in the analysis of clinical trial cohorts. Quantification of TF and comparison to real-world cohorts could characterize whether an enrolled population is representative of the expected clinical presentation of the disease. If a phase II trial has a high rate of disease stability without tumor response, measurement of ctDNA TF could identify whether these favorable outcomes may be due to the enrollment of patients with low ctDNA shed. Finally, some studies have found on subset analysis that those positive for certain mutations in ctDNA have a worse prognosis^32–34^; measurement of TF could clarify whether this prognostic effect is due to the expected behavior of patients with elevated TF or due to the specific variant being analyzed.

Another possible application for ctDNA TF is therapy selection in the context of multiple possible standard-of-care treatment options. For example, in cancers like aNSCLC where immunotherapy and chemo-immunotherapy represent alternate standard options without randomized trials available, a patient with a favorable prognosis based on low TF (and favorable immunotherapy biomarkers) may choose immunotherapy alone to avoid the toxicity of chemotherapy. Further validation of TF, as well as selection of an appropriate threshold to inform clinical decision making, should be undertaken through prospective trials. If a threshold is defined and validated, patients and providers may decide upon treatment plans with a lower expected toxicity for those with a good prognosis. Conversely, patients with elevated TF may need more aggressive treatment and could be candidates for new combination strategies, such as abiraterone plus androgen-deprivation therapy plus docetaxel,^35^ or those with high TF may choose to explore clinical trials earlier in their disease course. Importantly, additional work using a distinct ctDNA assay demonstrated that early change in the amount of ctDNA is associated with immunotherapy response in aNSCLC.^36^ In addition to the robust prognostic information, the potential predictive capacity of TF dynamics warrants further study.

It is critical to note that while evidence is accumulating on the prognostic role of TF, its predictive ability is still to be proved. Hence, whether these considerations will result in better outcomes (quality or quantity of life) for an individual patient if a different treatment course is taken based on TF remains an unknown counterfactual. One study prospectively evaluating TF-guided risk stratification is the ongoing PROTRACT study (NCT04015622), where mCRPC patients are randomized to either physician’s choice of therapy versus treatment directed by TF. In the TF arm, patients with <2% TF are offered second-generation hormonal therapy whereas chemotherapy is offered to those with TF >2%.

This research does have key limitations, predominantly from using observational evidence as its backbone. This creates a potential patient selection bias as providers may choose to send the liquid biopsy test only on those with or without certain traits, with good or bad expected outcomes, and/or only at a specific line of therapy. To what extent the population receiving liquid biopsy resembles the broader cancer population is a much bigger question (and one whose answer may change over time) and will need to be considered as further clinical validation of TF estimation is planned. Additionally, availability of TF was an entry requirement to our study whereas other covariables in our multivariable models could be missing, creating a potential bias favoring TF, although we have sought to mitigate this by only including variables with at least 80% completeness. Prospective validation in randomized studies that represent diverse patients is a way to overcome these limitations. For example, to truly validate TF as a new and independent prognostic variable would require incorporation of all variables in prior previously validated prognostic models for comparison with and without TF, optimal cut-offs defined for TF, and then a separate analysis with a validation cohort. This needs to be done for each cancer type. Whether the gains for any variable are marginal and whether it is cumbersome or costly to measure must also be considered. In the current study, we observed that providers frequently did not collect all elements of existing models^14–16^ in routine clinical care, so some potentially important covariables could not be included and detailed prognostic model benchmarking is instead planned as a future, prospective effort. This work was also limited to four common cancer types, so further validation should be pursued to understand applicability to other cancers. Additionally, some of the multivariable models included variables with low case/event counts for certain levels, especially for tumor types besides NSCLC. This is reflected in wider CIs in Figure 3 and while TF itself has adequate counts, multivariable models are interdependent and so future validation studies will need to be more adequately sized. While TF remained prognostic independent of line of therapy in each cancer type (Figure 3), further validation at specific lines of therapy (e.g. fourth line versus second line) would be valuable. In addition, further work on optimal TF cutpoint within each cancer type is warranted, including whether dichotomous, trichotomous, or other stratifications are most clinically useful. We did not measure radiographic tumor burden in this study, which is likely to correlate with ctDNA TF. However, radiographic measures of tumor burden can be technically variable across cancer types and across clinical care settings, making non-invasive quantification using ctDNA a potentially objective and accessible complement. Future advances in ctDNA assay development, including incorporation of ctDNA methylation features and fragment features, may further enhance the utility of TF measurements.

As circulating analytes continue to progress in their prognostic/predictive capacity, it is increasingly likely that a patient could have a circulating test done at the time of metastases to both (i) find alterations that could be exploited in current or subsequent therapy as well as (ii) allow risk stratification based on their oncologic trajectory.

## Supplementary Material

SupplementaryTables

SupplementaryFigures

## Figures and Tables

**Table 1.** Clinicopathological covariables

Tumor type	Variables used in modeling
**mCRPC**	Line number, practice type (community versus academic), age at therapy start, race, ECOG performance score, albumin below normal limits, alkaline phosphatase above normal limits, hemoglobin below normal limits, neutrophil-to-lymphocyte ratio, log2 prostate specific antigen
**mBC**	Line number, practice type (community versus academic), age at therapy start, race, ECOG performance score, hormone receptor status, Her2 status, sites of metastasis
**aNSCLC**	Line number, practice type (community versus academic), age at therapy start, race, ECOG performance score, sex, smoking status, EGFR mutation status, KRAS mutation status, BRAF mutation status, sites of metastasis, histology
**mCRC**	Line number, practice type (community versus academic), age at therapy start, race, ECOG performance score, sex, BRAF V600E mutation status, RAS mutation status, ERBB2 mutation status, sidedness at diagnosis

A panel of covariates was specific for each type of cancer. Variables were selected based on a combination of documented prognostic relevance in the tumor type and availability in the clinico-genomic database (CGDB). All variables were measured within the same 60-day pre-therapy window as TF except: hormone receptor and HER2 statuses in breast counted any pre-therapy positive result as positive for the patient, sites of metastasis counted all metastases detected pre-therapy, sidedness of CRC was indexed to initial diagnosis.

aNSCLC, advanced non-small-cell lung cancer; CRC, colorectal cancer; ECOG, Eastern Cooperative Oncology Group; EGFR, epidermal growth factor receptor; HER2, human epidermal growth factor receptor 2; mBC, metastatic breast cancer; mCRC, metastatic colorectal cancer; mCRPC, metastatic castration-resistant prostate cancer; TF, tumor fraction.
