# Supplementary material for: Prognostic value of plasma circulating tumor DNA fraction across four common cancer types: a real-world outcomes study
Source: Ann Oncol. Author manuscript; Available in PMC 2023 Jan 1. (PMC9805517; doi:10.1016/j.annonc.2022.09.163)
Supplement: SupplementaryFigures [file NIHMS1850389-supplement-SupplementaryFigures.pptx]

## Slide 1
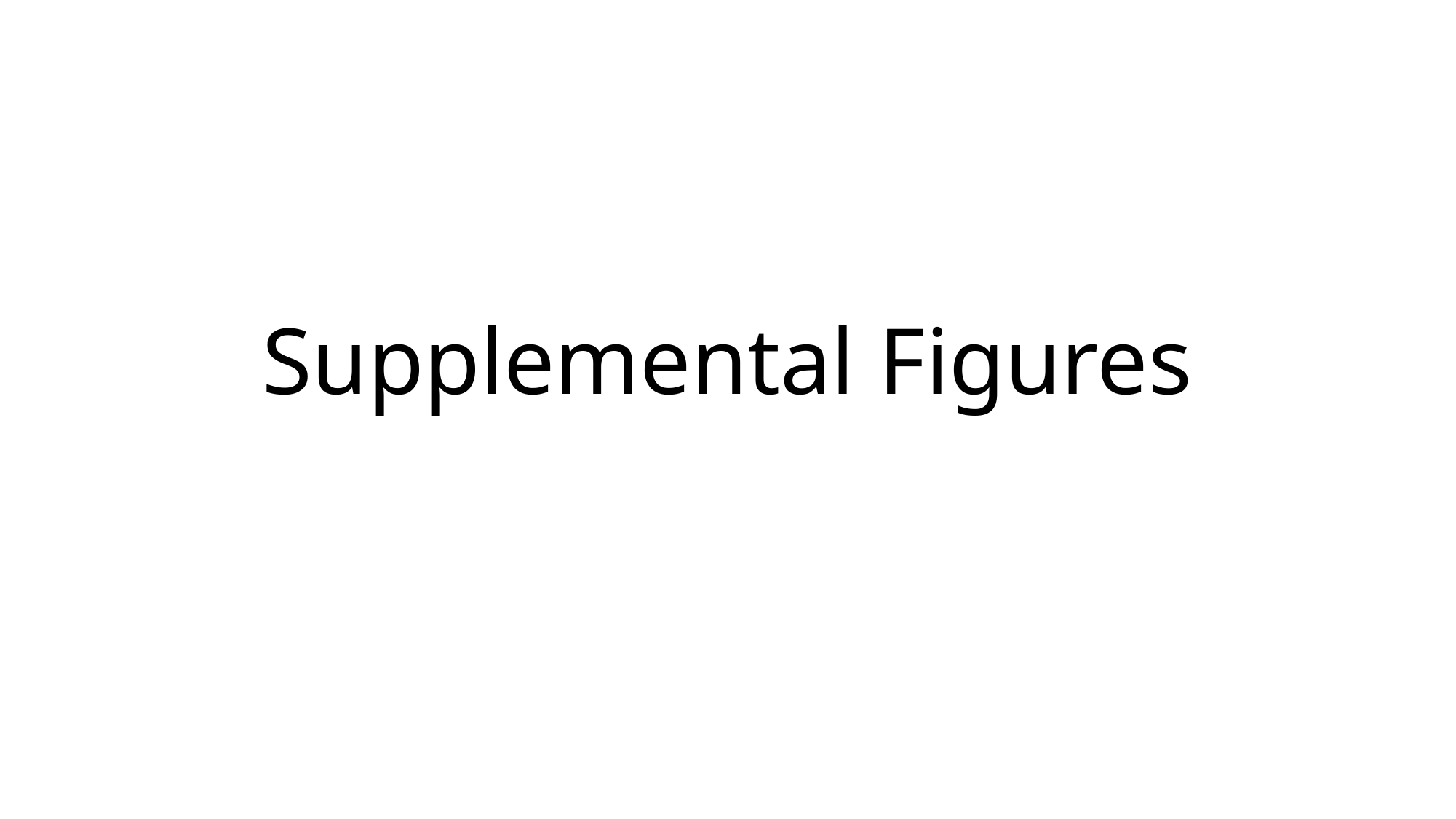

# Supplemental Figures

## Slide 2
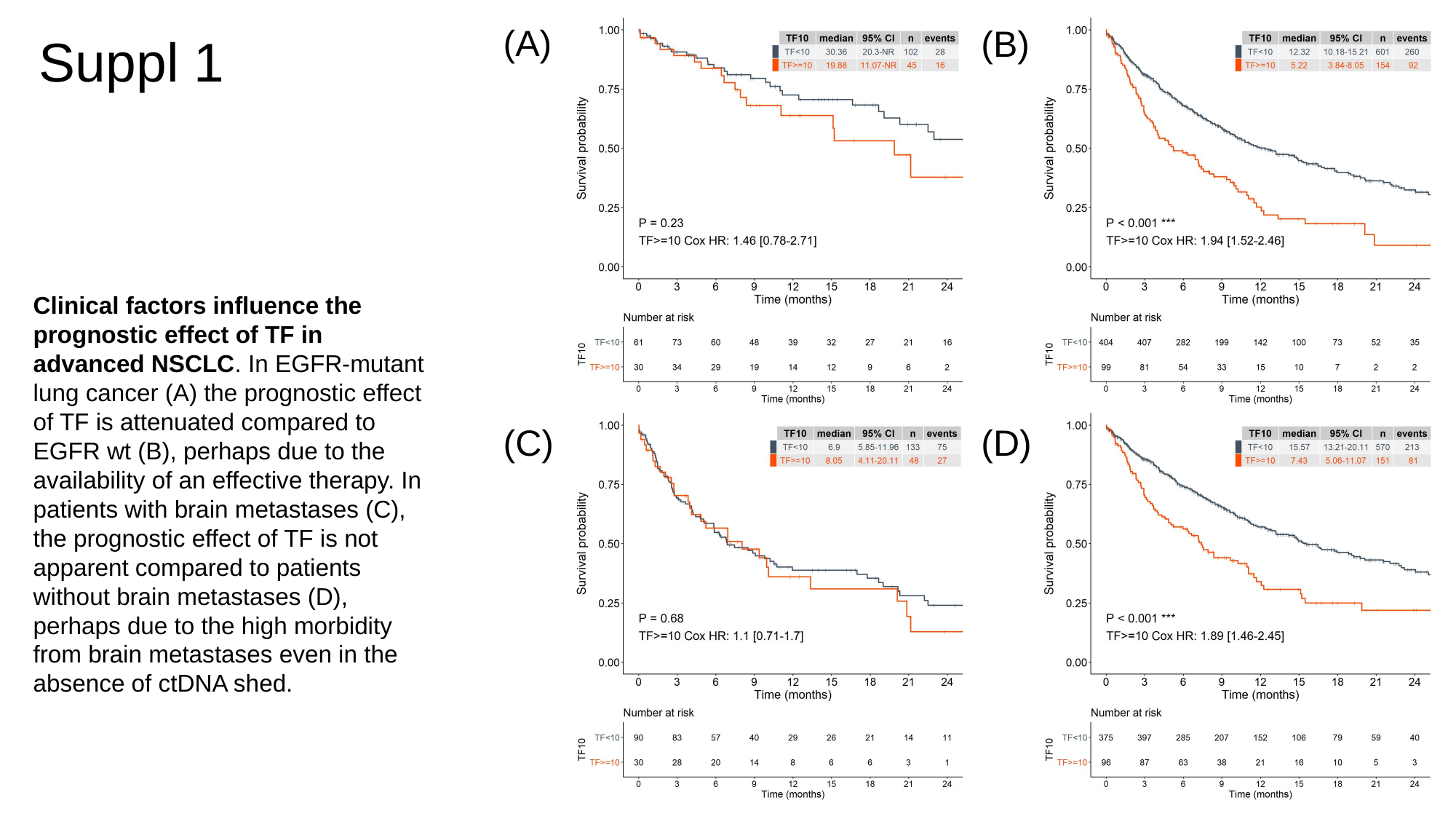

(A)
(B)
Suppl 1
Clinical factors influence the prognostic effect of TF in advanced NSCLC. In EGFR-mutant lung cancer (A) the prognostic effect of TF is attenuated compared to EGFR wt (B), perhaps due to the availability of an effective therapy. In patients with brain metastases (C), the prognostic effect of TF is not apparent compared to patients without brain metastases (D), perhaps due to the high morbidity from brain metastases even in the absence of ctDNA shed.
(C)
(D)

## Slide 3
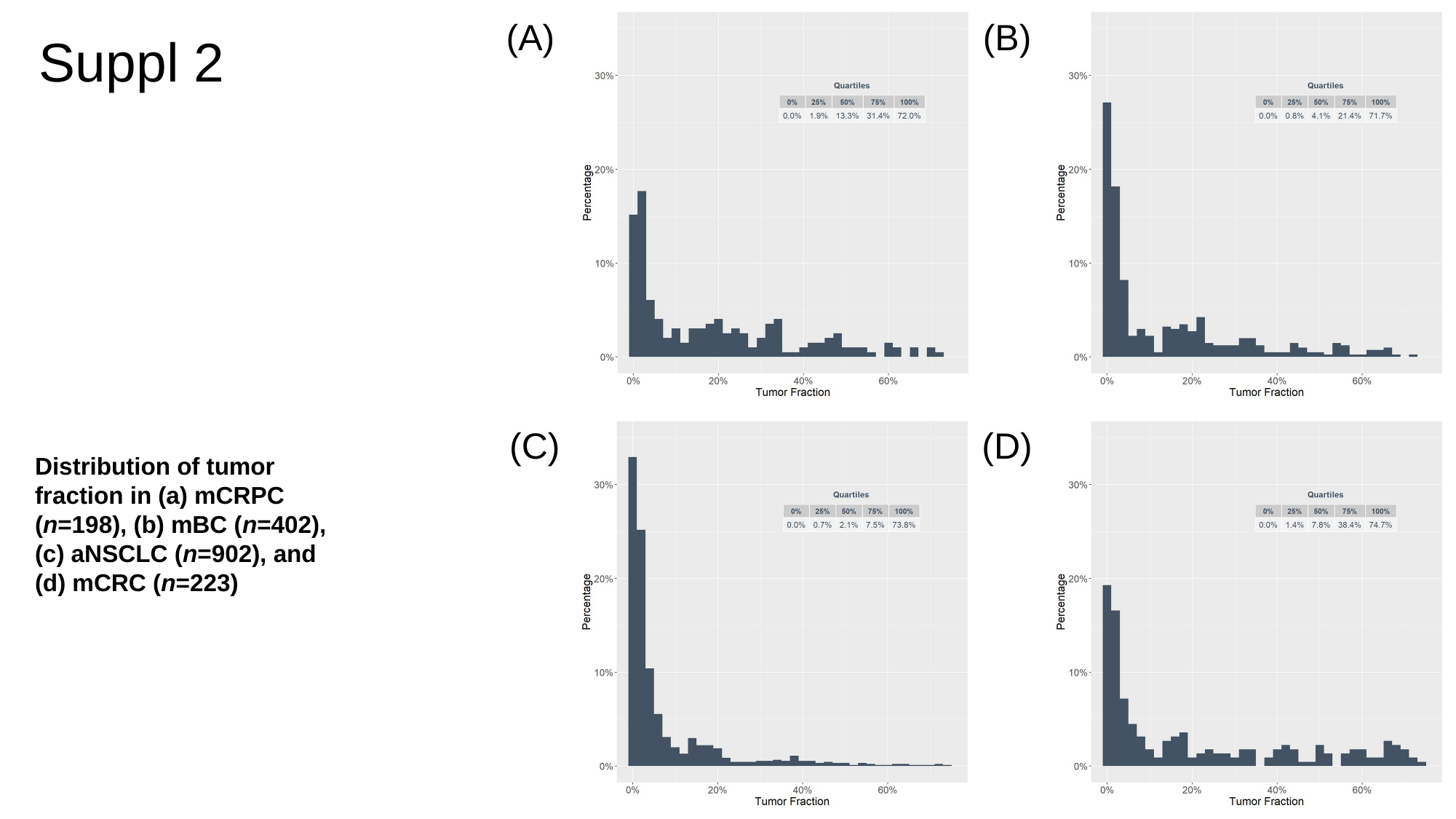

(A)
(B)
Suppl 2
(C)
(D)
Distribution of tumor fraction in (a) mCRPC (n=198), (b) mBC (n=402), (c) aNSCLC (n=902), and (d) mCRC (n=223)

## Slide 4
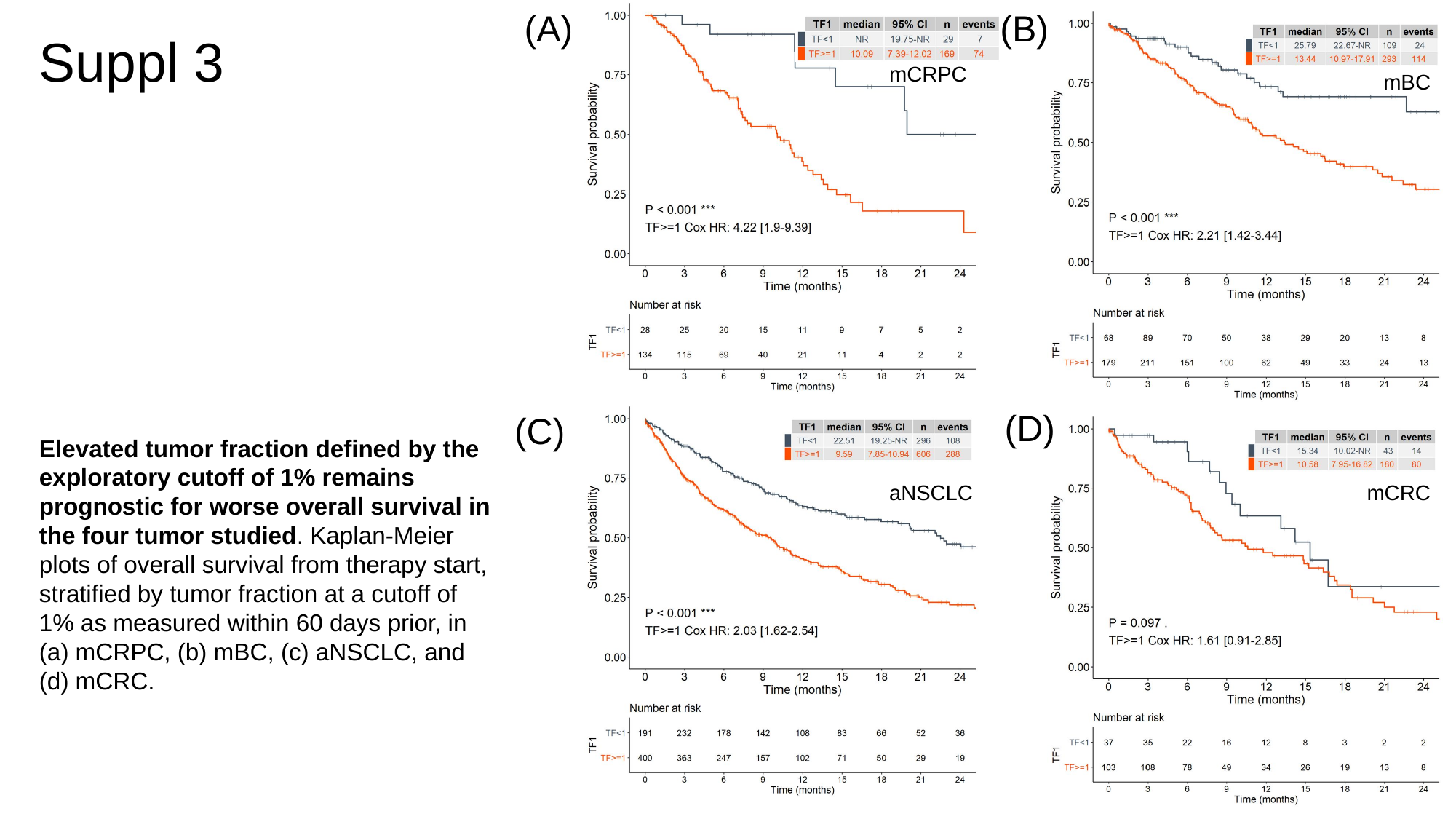

(A)
(B)
Suppl 3
mCRPC
mBC
(D)
(C)
Elevated tumor fraction defined by the exploratory cutoff of 1% remains prognostic for worse overall survival in the four tumor studied. Kaplan-Meier plots of overall survival from therapy start, stratified by tumor fraction at a cutoff of 1% as measured within 60 days prior, in (a) mCRPC, (b) mBC, (c) aNSCLC, and (d) mCRC.
aNSCLC
mCRC

## Slide 5
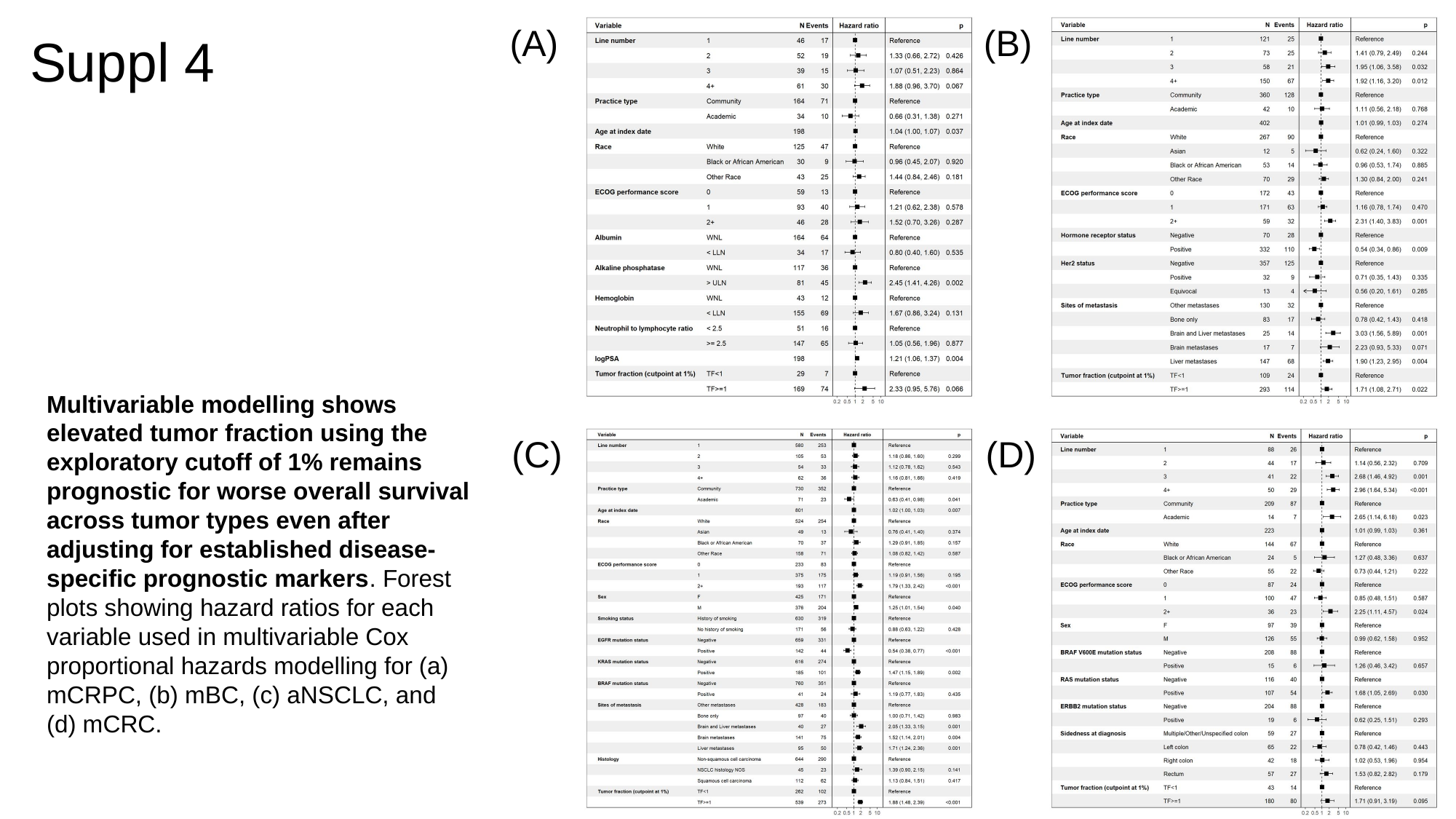

(A)
(B)
Suppl 4
Multivariable modelling shows elevated tumor fraction using the exploratory cutoff of 1% remains prognostic for worse overall survival across tumor types even after adjusting for established disease-specific prognostic markers. Forest plots showing hazard ratios for each variable used in multivariable Cox proportional hazards modelling for (a) mCRPC, (b) mBC, (c) aNSCLC, and (d) mCRC.
(C)
(D)

## Slide 6
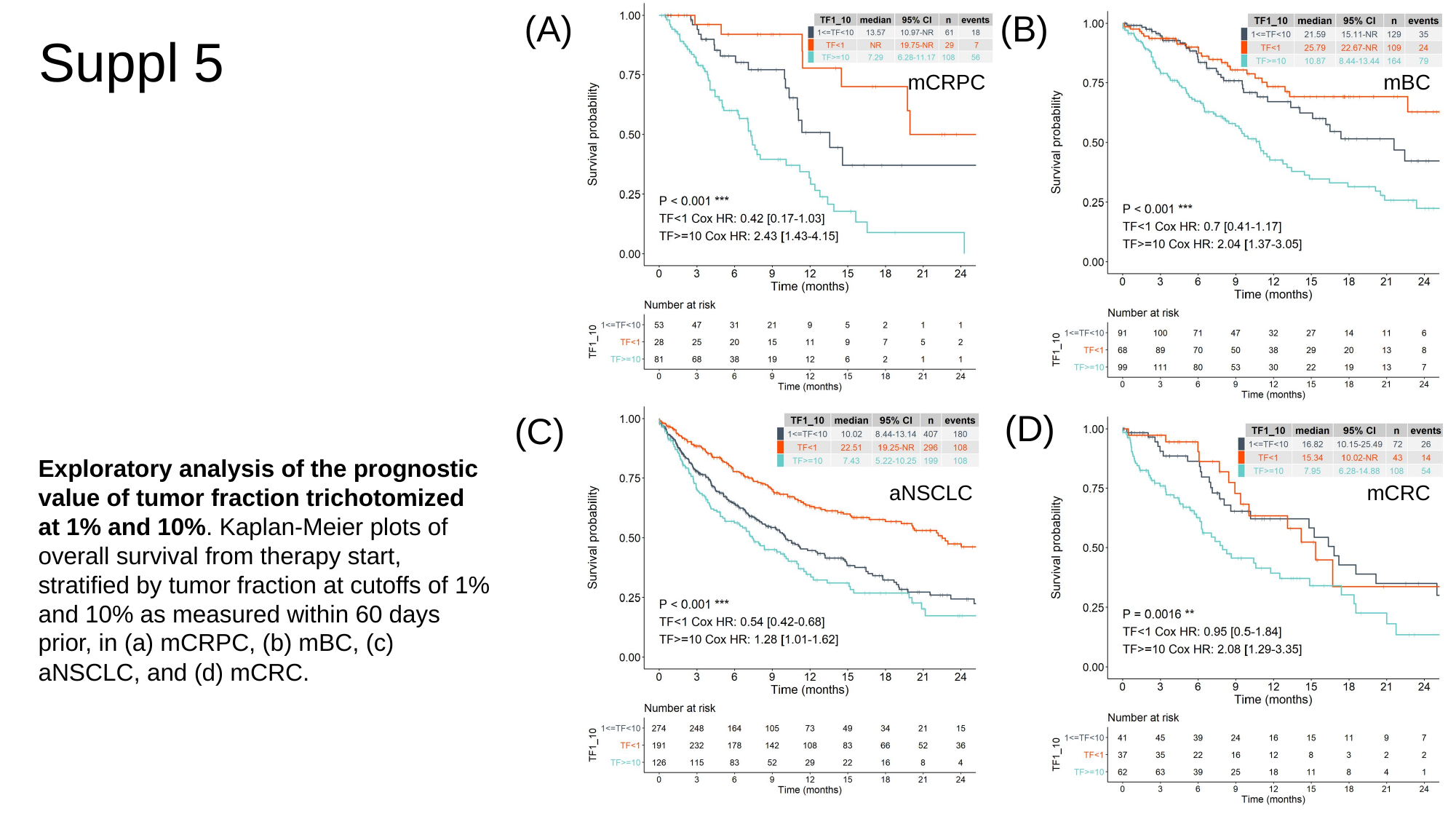

(A)
(B)
Suppl 5
mCRPC
mBC
(D)
(C)
Exploratory analysis of the prognostic value of tumor fraction trichotomized at 1% and 10%. Kaplan-Meier plots of overall survival from therapy start, stratified by tumor fraction at cutoffs of 1% and 10% as measured within 60 days prior, in (a) mCRPC, (b) mBC, (c) aNSCLC, and (d) mCRC.
aNSCLC
mCRC
